# Supplementary material for: Metabolic stress is a primary pathogenic event in transgenic Caenorhabditis elegans expressing pan-neuronal human amyloid beta
Source: eLife. 2019 Oct 15;8:e50069. doi: 10.7554/eLife.50069 (PMC6794093; doi:10.7554/eLife.50069)
Supplement: Supplementary file 1. [file elife-50069-supp1.docx]

**Supplementary file 1. Nutritional and metabolic parameters used in metabolic flux balance analysis.**

| Parameters | GRU101 | GRU102 | Ratio of GRU102/GRU101 |
| --- | --- | --- | --- |
| Oxygen uptake (umol/g.DW.h) | 734 | 837 | 1.13 |
| ATP production (umol/g.DW.h) | 3966 | 3280 | 0.826 |
| Pharyngeal pumping rate (pumps/min) | 380 | 306 | 0.805 |
| Body size (mm) | 1.42 | 1.27 | 0.894 |
| Biomass | 68.21 | 52.17 | 0.761 |
