# Supplementary material for: Metabolic stress is a primary pathogenic event in transgenic Caenorhabditis elegans expressing pan-neuronal human amyloid beta
Source: eLife. 2019 Oct 15;8:e50069. doi: 10.7554/eLife.50069 (PMC6794093; doi:10.7554/eLife.50069)
Supplement: Supplementary file 2. [file elife-50069-supp2.docx]

**Supplementary file 2. List of reactions that show directional inconsistency in flux variability analysis (FVA).**

| ***Reaction Name*** | ***Subsystems*** |
| --- | --- |
| xanthine:NAD+ oxidoreductase | Purine metabolism |
| ATP:CDP phosphotransferase | Pyrimidine metabolism |
| ATP:CMP phosphotransferase | Pyrimidine metabolism |
| L-Alanine:2-oxoglutarate aminotransferase | Alanine, aspartate and glutamate metabolism |
| Hydrogen peroxide transport | Transport |
| L-Serine transport | Transport |
| Coenzyme A transport | Transport |
| H2O exchange | Exchange with the environment |
