# Supplementary material for: Metabolic stress is a primary pathogenic event in transgenic Caenorhabditis elegans expressing pan-neuronal human amyloid beta
Source: eLife. 2019 Oct 15;8:e50069. doi: 10.7554/eLife.50069 (PMC6794093; doi:10.7554/eLife.50069)
Supplement: Supplementary file 3. [file elife-50069-supp3.docx]

**Supplementary file 3. Summary of all lifespan trials with Metformin-treatment.**

|  | Median LS (no of animals) | | | Log-rank test *p* value | | |
| --- | --- | --- | --- | --- | --- | --- |
|  | GRU101 | GRU102 | GRU102 + 50mM Metformin | GRU101 vs GRU102 | GRU102 vs GRU102 + 50mM Metformin | GRU101 vs 50mM Metformin |
| Trial 1 | 18 (90) | 14 (73) | 18 (68) | P<0.001 | P<0.001 | ns |
| Trial 2 | 17 (74) | 14 (86) | 19 (74) | P<0.001 | P<0.005 | ns |
| Trial 3 | 18 (69) | 15 (87) | 18 (79) | P<0.001 | P<0.005 | ns |
